# Supplementary material for: Mechanism for microbial population collapse in a fluctuating resource environment
Source: Mol Syst Biol. 2017 Mar 20;13(3):919. doi: 10.15252/msb.20167058 (PMC5371734; doi:10.15252/msb.20167058)
Supplement: Supplementary file 1 — Appendix [file MSB-13-919-s001.pdf]

APPENDIX

Mechanism for microbial population collapse in a fluctuating resource environment

Serdar Turkarslan, Arjun V Raman, Anne W Thompson, Christina E Arens, Mark A Gillespie, Frederick von Netzer, Kristina L Hillesland, Sergey Stolyar, Adrian López García de Lomana, David J Reiss, Drew Gorman-Lewis, Grant M Zane, Jeffrey A Ranish, Judy D Wall, David A Stahl and Nitin S Baliga

Appendix Table of Contents

Figure Legends: Appendix Figure S1-S2 **Page 2**

Appendix Figure S1 **Page 3**

Appendix Figure S2 **Page 13**

Appendix Figure Legends

Appendix Figure S1: Change in abundance of selected Dv transcripts during experimental evolution of wild type and mutant co-cultures. Abundance for each transcript is represented as normalized read counts on the y-axis while conditions are listed on the x-axis.

Appendix Figure S2: Change in abundance (log_2) of selected Dv proteins during experimental evolution of wild type and mutant co-cultures. Protein abundances for each condition are listed as boxplots while trends are represented with a trend line. Log_2-fold changes between ST1 and SR3 were calculated by Student's t-test or Mann–Whitney U test depending on normality of observed values; normality assessed by Shapiro–Wilk normality test. * indicates p-value < 0.05 or ** indicates p-value < 0.01. Essential gene names are indicated with red font.

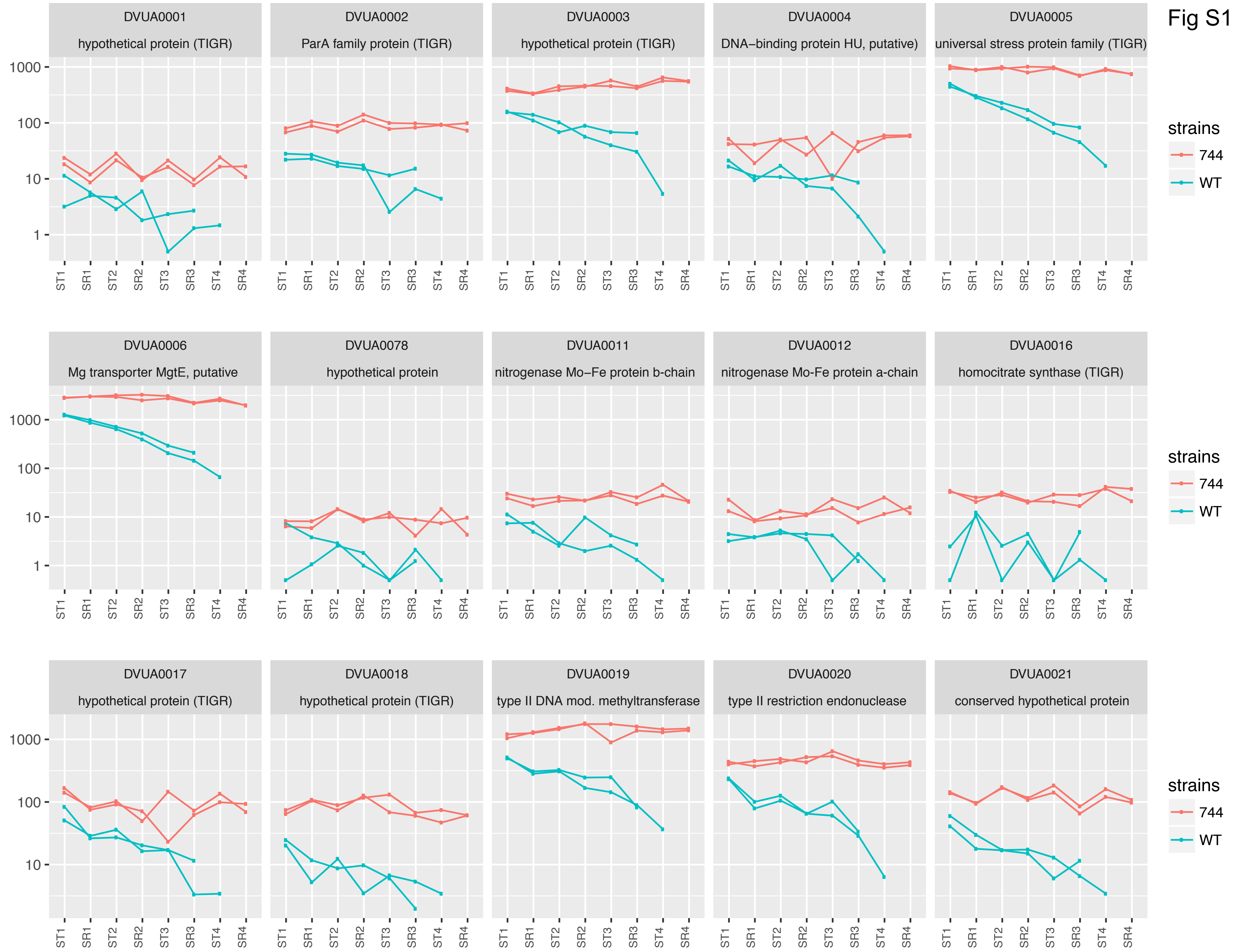
Fig S1
DVUA0001
hypothetical protein (TIGR)
DVUA0002
ParA family protein (TIGR)
DVUA0003
hypothetical protein (TIGR)
DVUA0004
DNA–binding protein HU, putative)
DVUA0005
universal stress protein family (TIGR)
DVUA0006
Mg transporter MgtE, putative
DVUA0078
hypothetical protein
DVUA0011
nitrogenase Mo–Fe protein b-chain
DVUA0012
nitrogenase Mo-Fe protein a-chain
DVUA0016
homocitrate synthase (TIGR)
DVUA0017
hypothetical protein (TIGR)
DVUA0018
hypothetical protein (TIGR)
DVUA0019
type II DNA mod. methyltransferase
DVUA0020
type II restriction endonuclease
DVUA0021
conserved hypothetical protein
1000
100
10
1
ST1
SR1
ST2
SR2
ST3
SR3
ST4
SR4
strains
744
WT

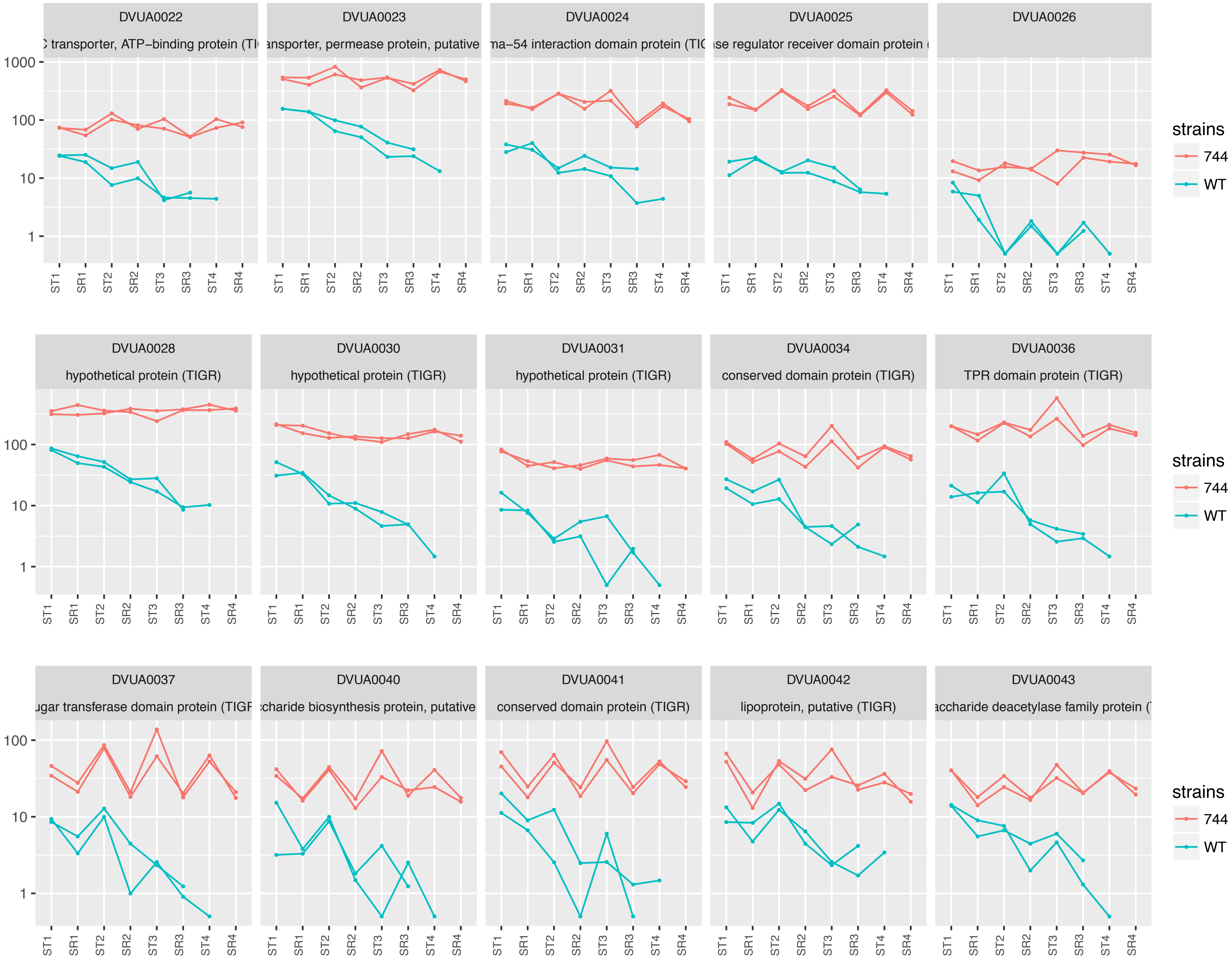
DVUA0022
C transporter, ATP–binding protein (TI
DVUA0023
ansporter, permease protein, putative
DVUA0024
ma–54 interaction domain protein (TIG
DVUA0025
se regulator receiver domain protein
DVUA0026
DVUA0028
hypothetical protein (TIGR)
DVUA0030
hypothetical protein (TIGR)
DVUA0031
hypothetical protein (TIGR)
DVUA0034
conserved domain protein (TIGR)
DVUA0036
TPR domain protein (TIGR)
DVUA0037
ugar transferase domain protein (TIGF
DVUA0040
charide biosynthesis protein, putative
DVUA0041
conserved domain protein (TIGR)
DVUA0042
lipoprotein, putative (TIGR)
DVUA0043
accharide deacetylase family protein (
1000
100
10
1
ST1
SR1
ST2
SR2
ST3
SR3
ST4
SR4
strains
744
WT

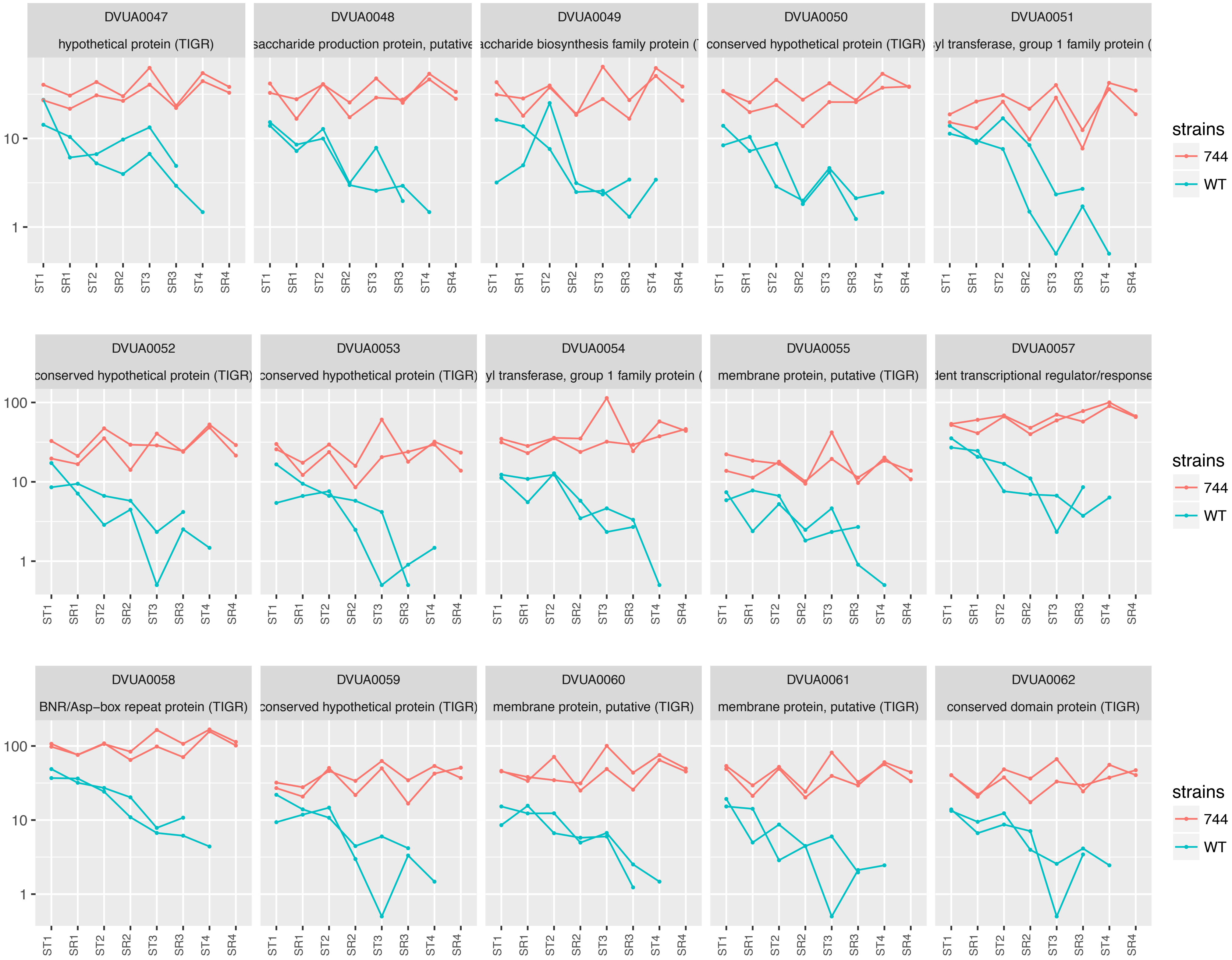
DVUA0047
hypothetical protein (TIGR)
DVUA0048
saccharide production protein, putative
DVUA0049
accharide biosynthesis family protein (
DVUA0050
conserved hypothetical protein (TIGR)
DVUA0051
yl transferase, group 1 family protein (
DVUA0052
conserved hypothetical protein (TIGR)
DVUA0053
conserved hypothetical protein (TIGR)
DVUA0054
yl transferase, group 1 family protein (
DVUA0055
membrane protein, putative (TIGR)
DVUA0057
dent transcriptional regulator/response
DVUA0058
BNR/Asp−box repeat protein (TIGR)
DVUA0059
conserved hypothetical protein (TIGR)
DVUA0060
membrane protein, putative (TIGR)
DVUA0061
membrane protein, putative (TIGR)
DVUA0062
conserved domain protein (TIGR)
100
10
1
ST1
SR1
ST2
SR2
ST3
SR3
ST4
SR4
strains
744
WT

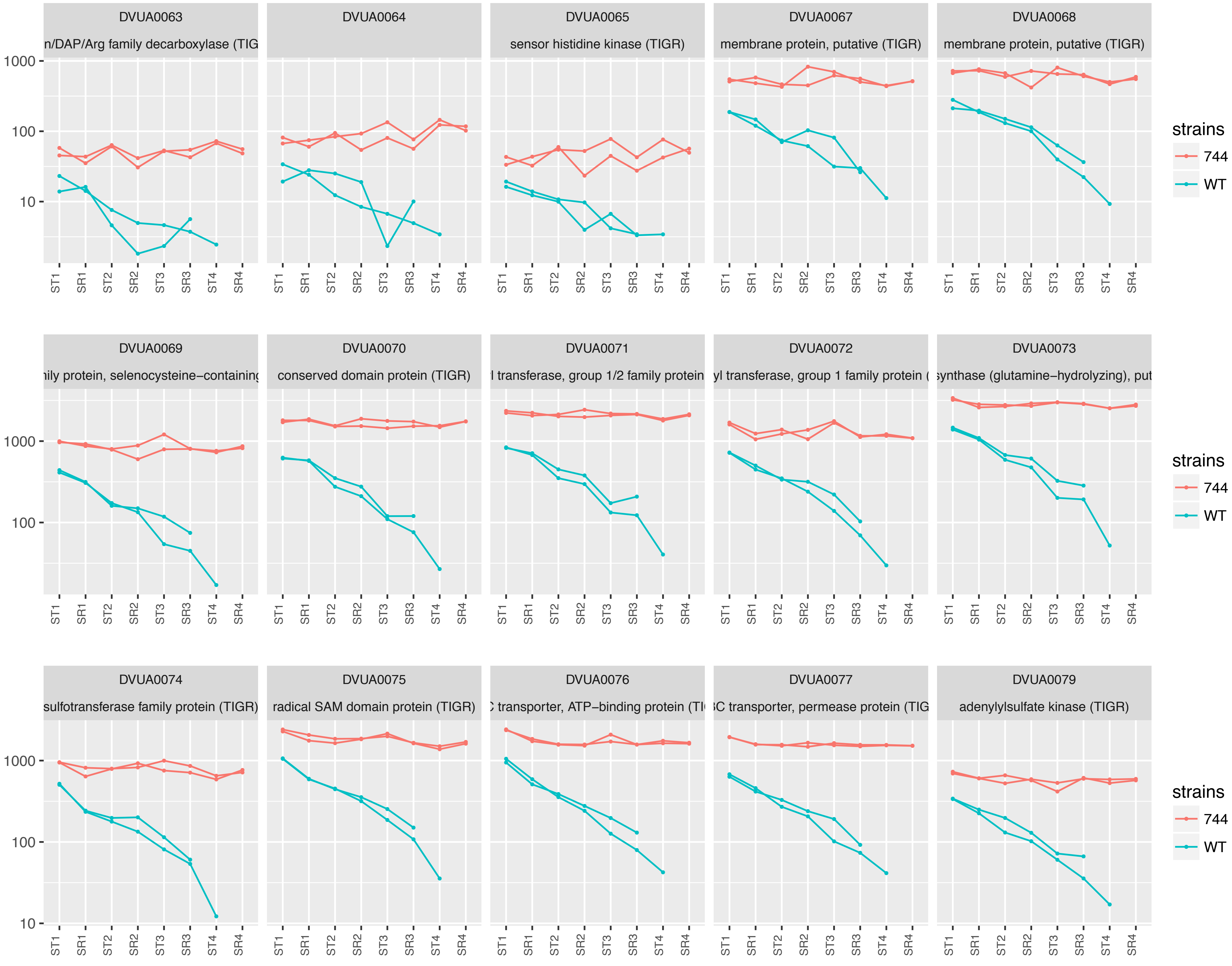
DVUA0063
n/DAP/Arg family decarboxylase (TIG
DVUA0064
DVUA0065
sensor histidine kinase (TIGR)
DVUA0067
membrane protein, putative (TIGR)
DVUA0068
membrane protein, putative (TIGR)
DVUA0069
ily protein, selenocysteine−containing
DVUA0070
conserved domain protein (TIGR)
DVUA0071
l transferase, group 1/2 family protein
DVUA0072
yl transferase, group 1 family protein (
DVUA0073
synthase (glutamine−hydrolyzing), put
DVUA0074
sulfotransferase family protein (TIGR)
DVUA0075
radical SAM domain protein (TIGR)
DVUA0076
C transporter, ATP−binding protein (TI
DVUA0077
C transporter, permease protein (TIG
DVUA0079
adenylylsulfate kinase (TIGR)
1000
100
10
ST1
SR1
ST2
SR2
ST3
SR3
ST4
SR4
strains
744
WT

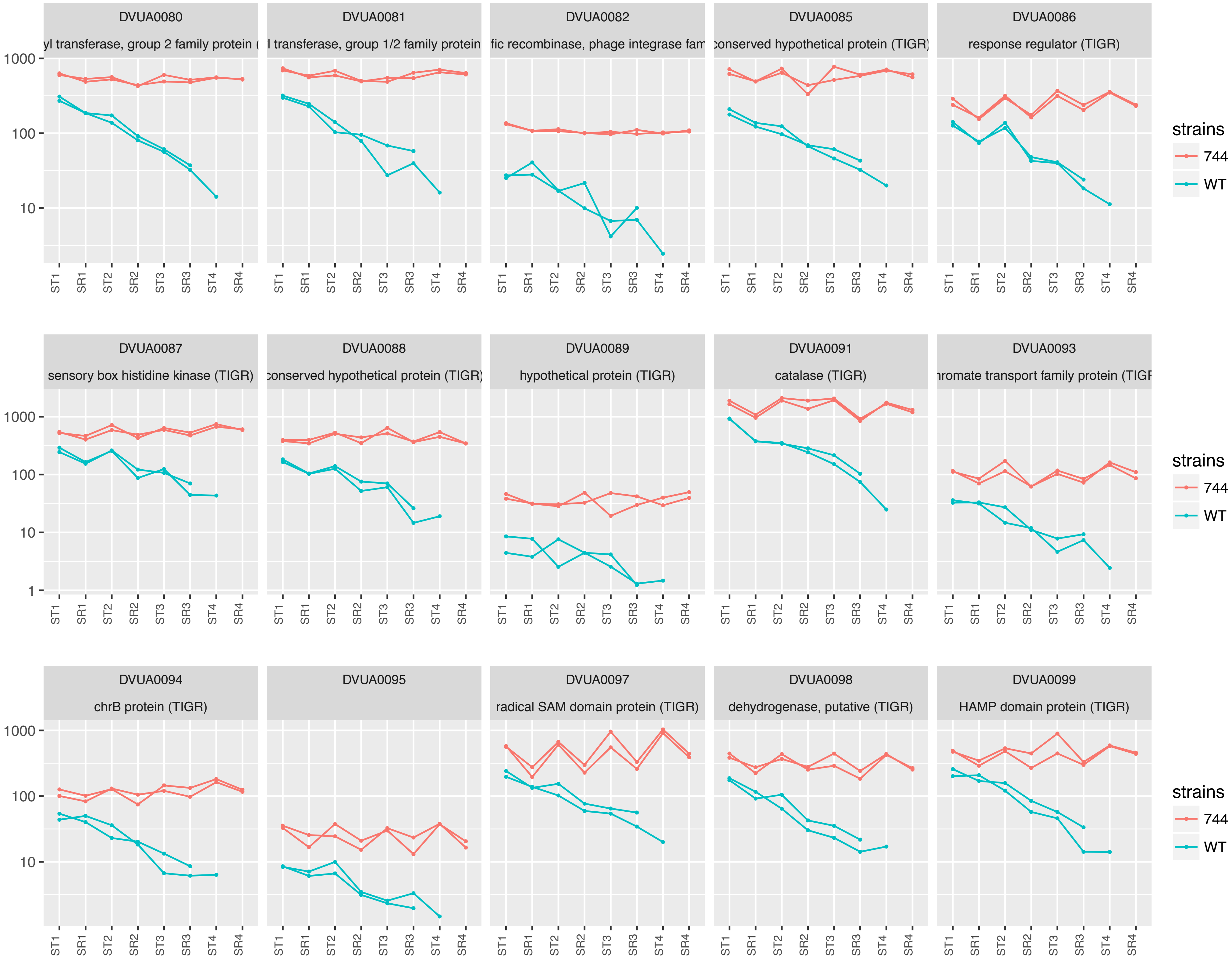

DVUA0080
yl transferase, group 2 family protein (
DVUA0081
l transferase, group 1/2 family protein
DVUA0082
fic recombinase, phage integrase fam
DVUA0085
conserved hypothetical protein (TIGR)
DVUA0086
response regulator (TIGR)
DVUA0087
sensory box histidine kinase (TIGR)
DVUA0088
conserved hypothetical protein (TIGR)
DVUA0089
hypothetical protein (TIGR)
DVUA0091
catalase (TIGR)
DVUA0093
hromate transport family protein (TIGF
DVUA0094
chrB protein (TIGR)
DVUA0095
DVUA0097
radical SAM domain protein (TIGR)
DVUA0098
dehydrogenase, putative (TIGR)
DVUA0099
HAMP domain protein (TIGR)
1
10
100
1000
ST1 SR1 ST2 SR2 ST3 SR3 ST4 SR4
strains
744
WT

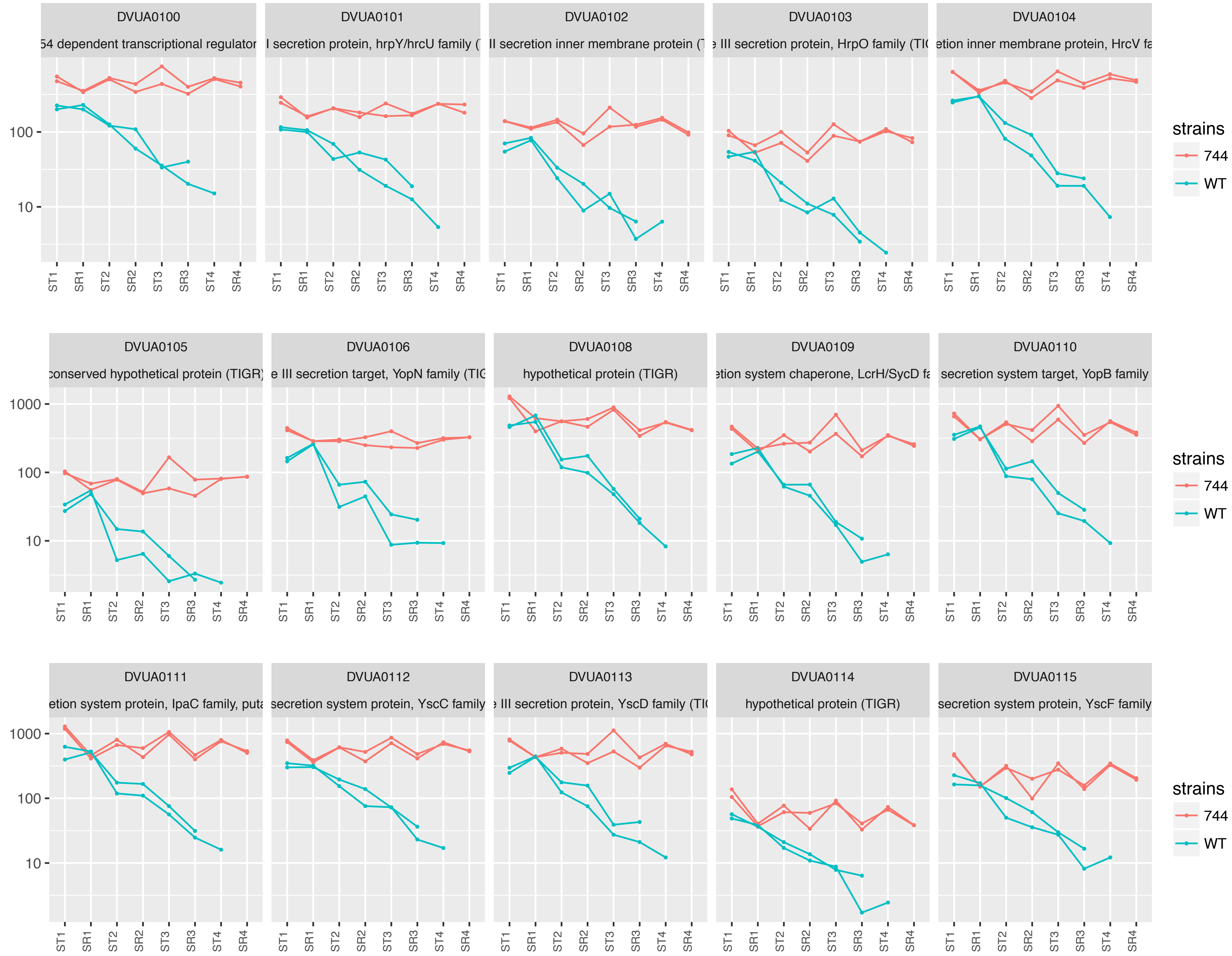
DVUA0100
54 dependent transcriptional regulator
DVUA0101
I secretion protein, hrpY/hrcU family (
DVUA0102
II secretion inner membrane protein (T
DVUA0103
e III secretion protein, HrpO family (TI(
DVUA0104
etion inner membrane protein, HrcV fa
DVUA0105
conserved hypothetical protein (TIGR)
DVUA0106
e III secretion target, YopN family (TIG
DVUA0108
hypothetical protein (TIGR)
DVUA0109
etion system chaperone, LcrH/SycD fa
DVUA0110
secretion system target, YopB family
DVUA0111
etion system protein, IpaC family, puta
DVUA0112
secretion system protein, YscC family
DVUA0113
e III secretion protein, YscD family (TI(
DVUA0114
hypothetical protein (TIGR)
DVUA0115
secretion system protein, YscF family
1000
100
10
ST1
SR1
ST2
SR2
ST3
SR3
ST4
SR4
strains
744
WT

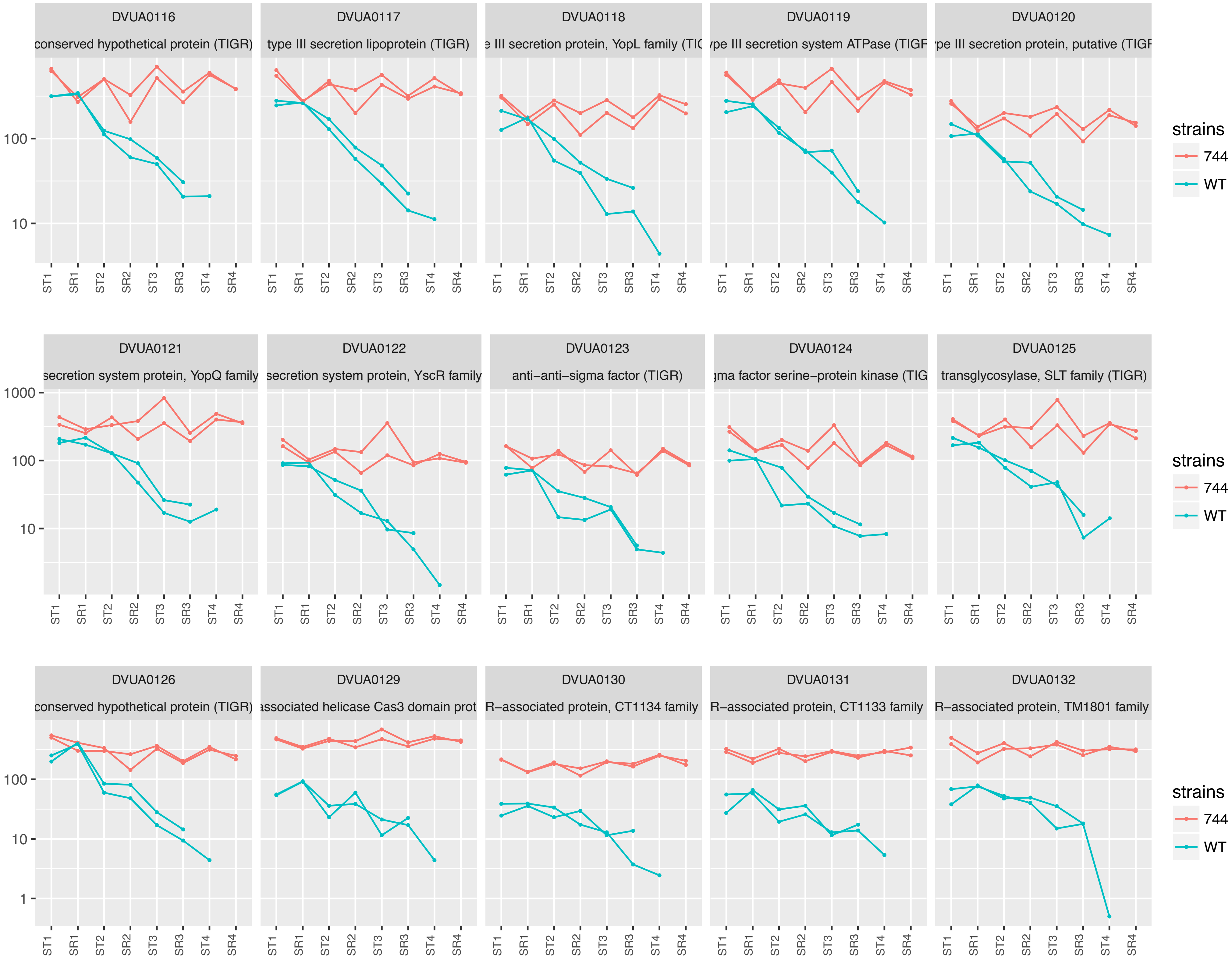

DVUA0116
conserved hypothetical protein (TIGR)
DVUA0117
type III secretion lipoprotein (TIGR)
DVUA0118
e III secretion protein, YopL family (TIG
DVUA0119
ype III secretion system ATPase (TIGF
DVUA0120
ype III secretion protein, putative (TIGF
DVUA0121
secretion system protein, YopQ family
DVUA0122
secretion system protein, YscR family
DVUA0123
anti–anti–sigma factor (TIGR)
DVUA0124
gma factor serine–protein kinase (TIG
DVUA0125
transglycosylase, SLT family (TIGR)
DVUA0126
conserved hypothetical protein (TIGR)
DVUA0129
associated helicase Cas3 domain prot
DVUA0130
R–associated protein, CT1134 family
DVUA0131
R–associated protein, CT1133 family
DVUA0132
R–associated protein, TM1801 family
1000
100
10
1
ST1
SR1
ST2
SR2
ST3
SR3
ST4
SR4
strains
744
WT

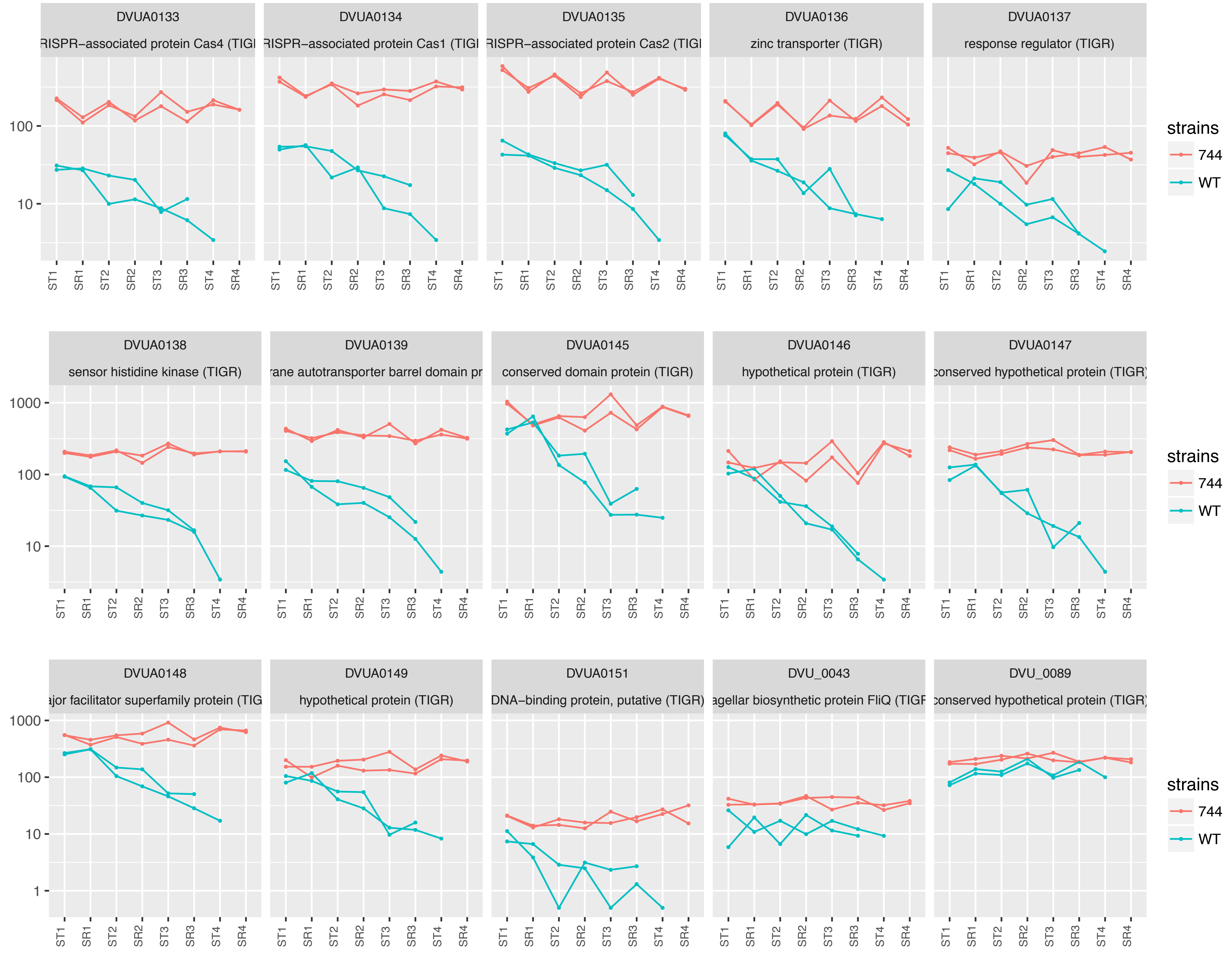
DVUA0133
RISPR–associated protein Cas4 (TIGI
DVUA0134
RISPR–associated protein Cas1 (TIGI
DVUA0135
RISPR–associated protein Cas2 (TIGI
DVUA0136
zinc transporter (TIGR)
DVUA0137
response regulator (TIGR)
DVUA0138
sensor histidine kinase (TIGR)
DVUA0139
rane autotransporter barrel domain pr
DVUA0145
conserved domain protein (TIGR)
DVUA0146
hypothetical protein (TIGR)
DVUA0147
conserved hypothetical protein (TIGR)
DVUA0148
ajor facilitator superfamily protein (TIG
DVUA0149
hypothetical protein (TIGR)
DVUA0151
DNA–binding protein, putative (TIGR)
DVU_0043
agellar biosynthetic protein FliQ (TIGF
DVU_0089
conserved hypothetical protein (TIGR)
1000
100
10
1
ST1
SR1
ST2
SR2
ST3
SR3
ST4
SR4
strains
744
WT

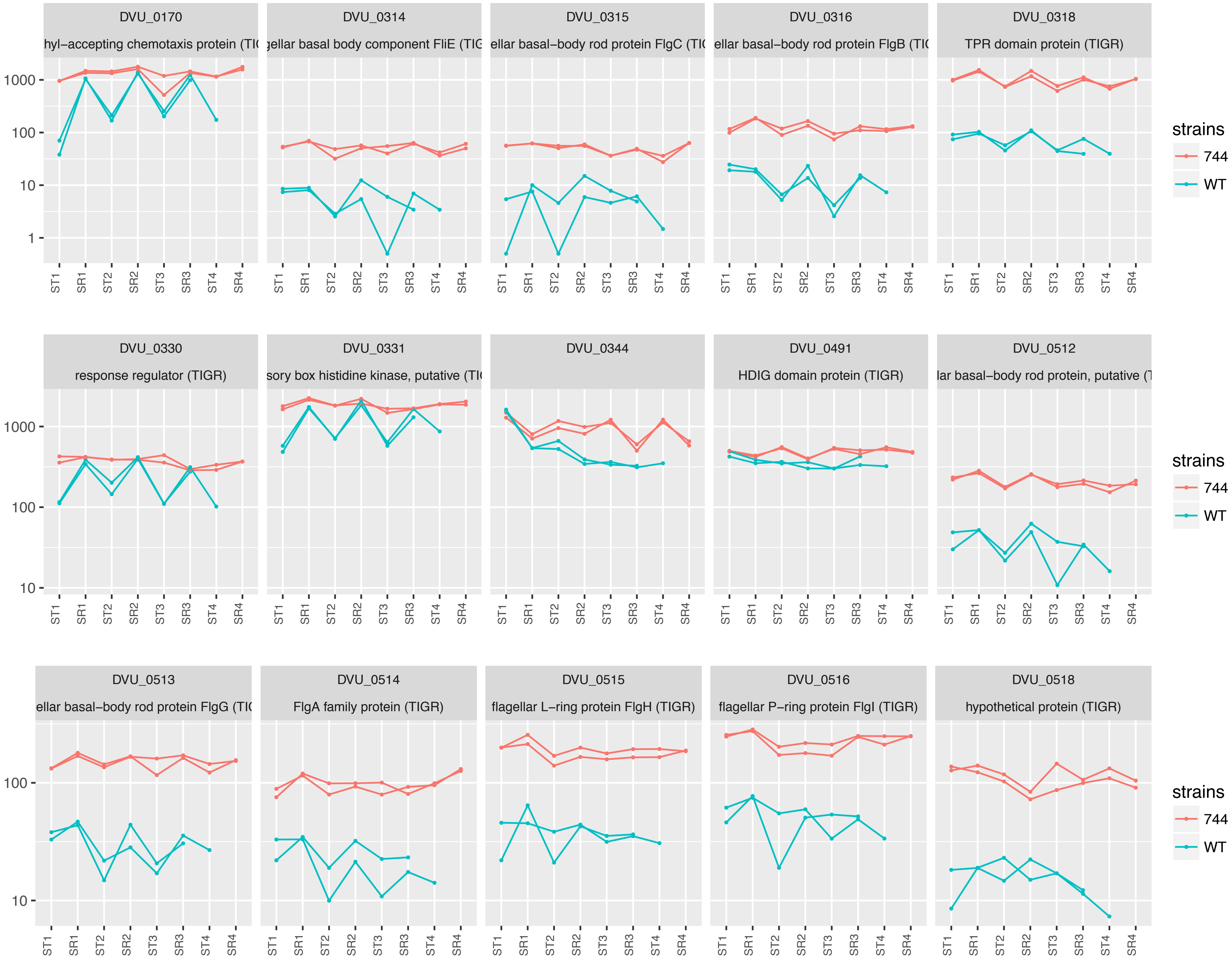
DVU_0170
hyl–accepting chemotaxis protein (TI(
DVU_0314
gellar basal body component FliE (TIG
DVU_0315
ellar basal–body rod protein FlgC (TI(
DVU_0316
ellar basal–body rod protein FlgB (TI(
DVU_0318
TPR domain protein (TIGR)
DVU_0330
response regulator (TIGR)
DVU_0331
sory box histidine kinase, putative (TI(
DVU_0344
DVU_0491
HDIG domain protein (TIGR)
DVU_0512
lar basal–body rod protein, putative (T
DVU_0513
ellar basal–body rod protein FlgG (TI(
DVU_0514
FlgA family protein (TIGR)
DVU_0515
flagellar L–ring protein FlgH (TIGR)
DVU_0516
flagellar P–ring protein FlgI (TIGR)
DVU_0518
hypothetical protein (TIGR)
1000
100
10
1
ST1
SR1
ST2
SR2
ST3
SR3
ST4
SR4
strains
744
WT

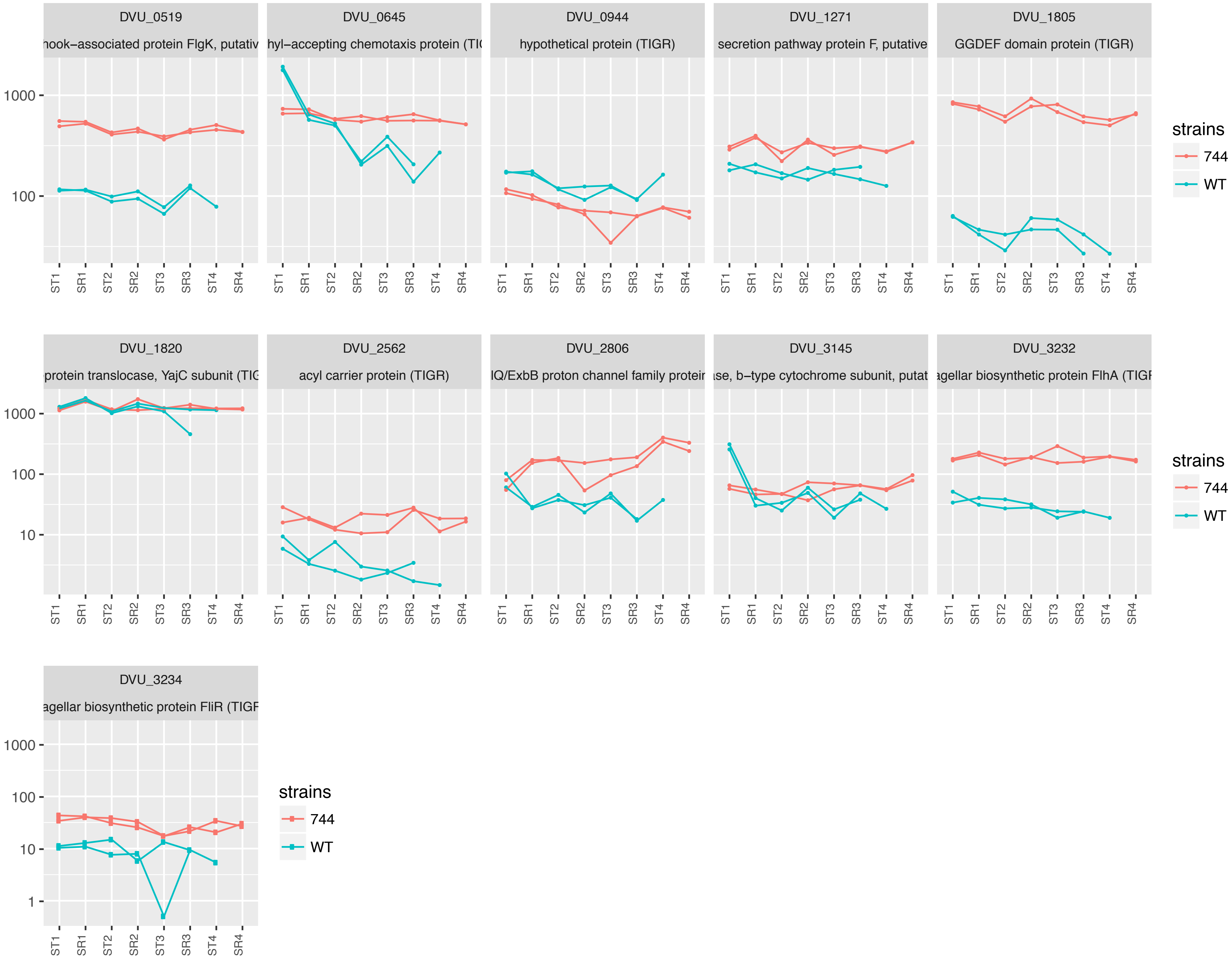
DVU_0519
hook–associated protein FlgK, putativ
DVU_0645
hyl–accepting chemotaxis protein (TI
DVU_0944
hypothetical protein (TIGR)
DVU_1271
secretion pathway protein F, putative
DVU_1805
GGDEF domain protein (TIGR)
DVU_1820
protein translocase, YajC subunit (TIG
DVU_2562
acyl carrier protein (TIGR)
DVU_2806
lQ/ExbB proton channel family proteir
DVU_3145
ıse, b–type cytochrome subunit, putat
DVU_3232
agellar biosynthetic protein FlhA (TIGF
DVU_3234
agellar biosynthetic protein FliR (TIGF
1000
100
10
1
ST1
SR1
ST2
SR2
ST3
SR3
ST4
SR4
strains
744
WT

Fig S2

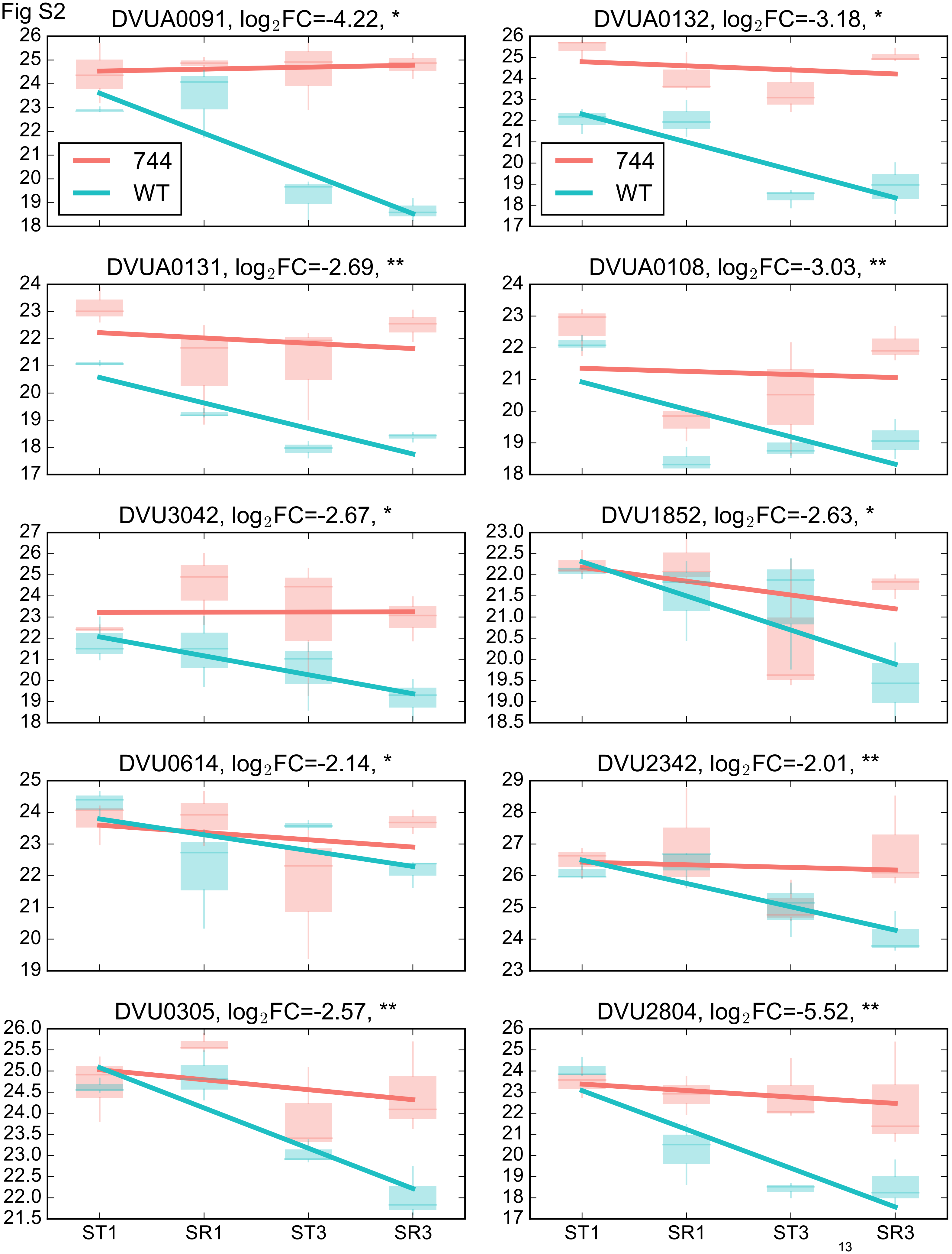
DVUA0091, log$_2$FC=-4.22, *
DVUA0132, log$_2$FC=-3.18, *
DVUA0131, log$_2$FC=-2.69, **
DVUA0108, log$_2$FC=-3.03, **
DVU3042, log$_2$FC=-2.67, *
DVU1852, log$_2$FC=-2.63, *
DVU0614, log$_2$FC=-2.14, *
DVU2342, log$_2$FC=-2.01, **
DVU0305, log$_2$FC=-2.57, **
DVU2804, log$_2$FC=-5.52, **
744
WT
ST1
SR1
ST3
SR3

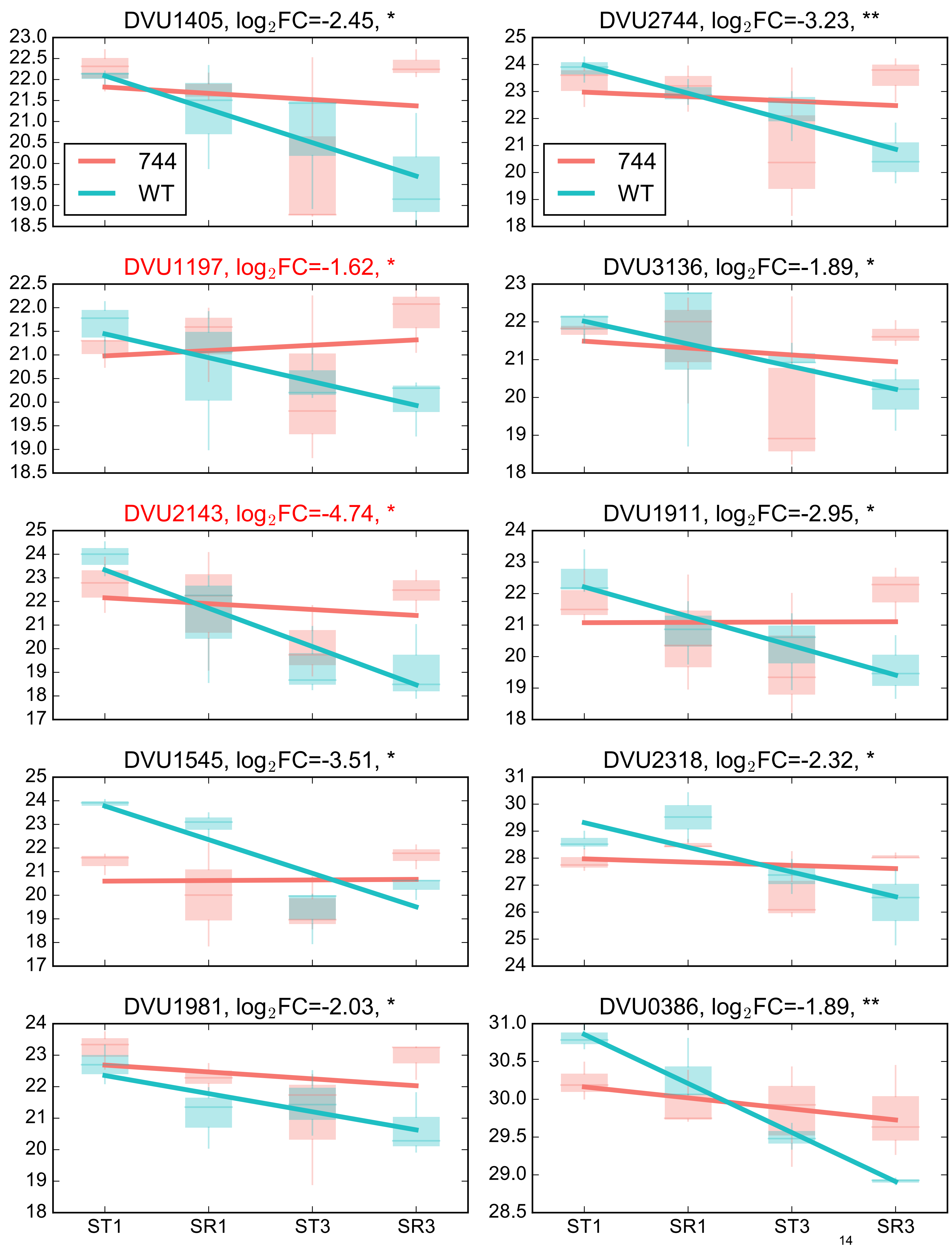
DVU1405, log_2FC=-2.45, *
DVU2744, log_2FC=-3.23, **
744
WT
DVU1197, log_2FC=-1.62, *
DVU3136, log_2FC=-1.89, *
DVU2143, log_2FC=-4.74, *
DVU1911, log_2FC=-2.95, *
DVU1545, log_2FC=-3.51, *
DVU2318, log_2FC=-2.32, *
DVU1981, log_2FC=-2.03, *
DVU0386, log_2FC=-1.89, **
ST1
SR1
ST3
SR3

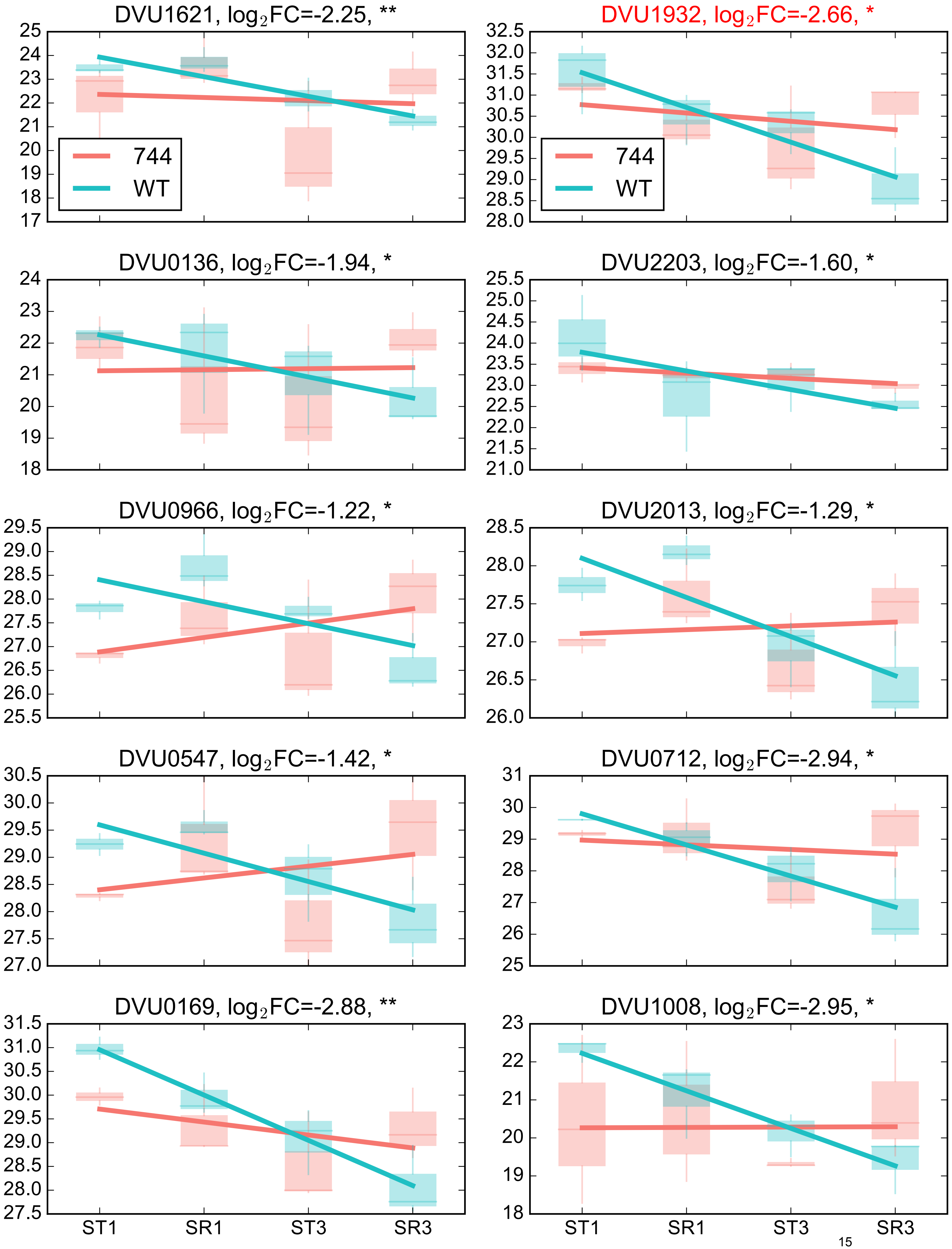
DVU1621, log2FC=-2.25, **
DVU1932, log2FC=-2.66, *
DVU0136, log2FC=-1.94, *
DVU2203, log2FC=-1.60, *
DVU0966, log2FC=-1.22, *
DVU2013, log2FC=-1.29, *
DVU0547, log2FC=-1.42, *
DVU0712, log2FC=-2.94, *
DVU0169, log2FC=-2.88, **
DVU1008, log2FC=-2.95, *
744
WT
ST1
SR1
ST3
SR3

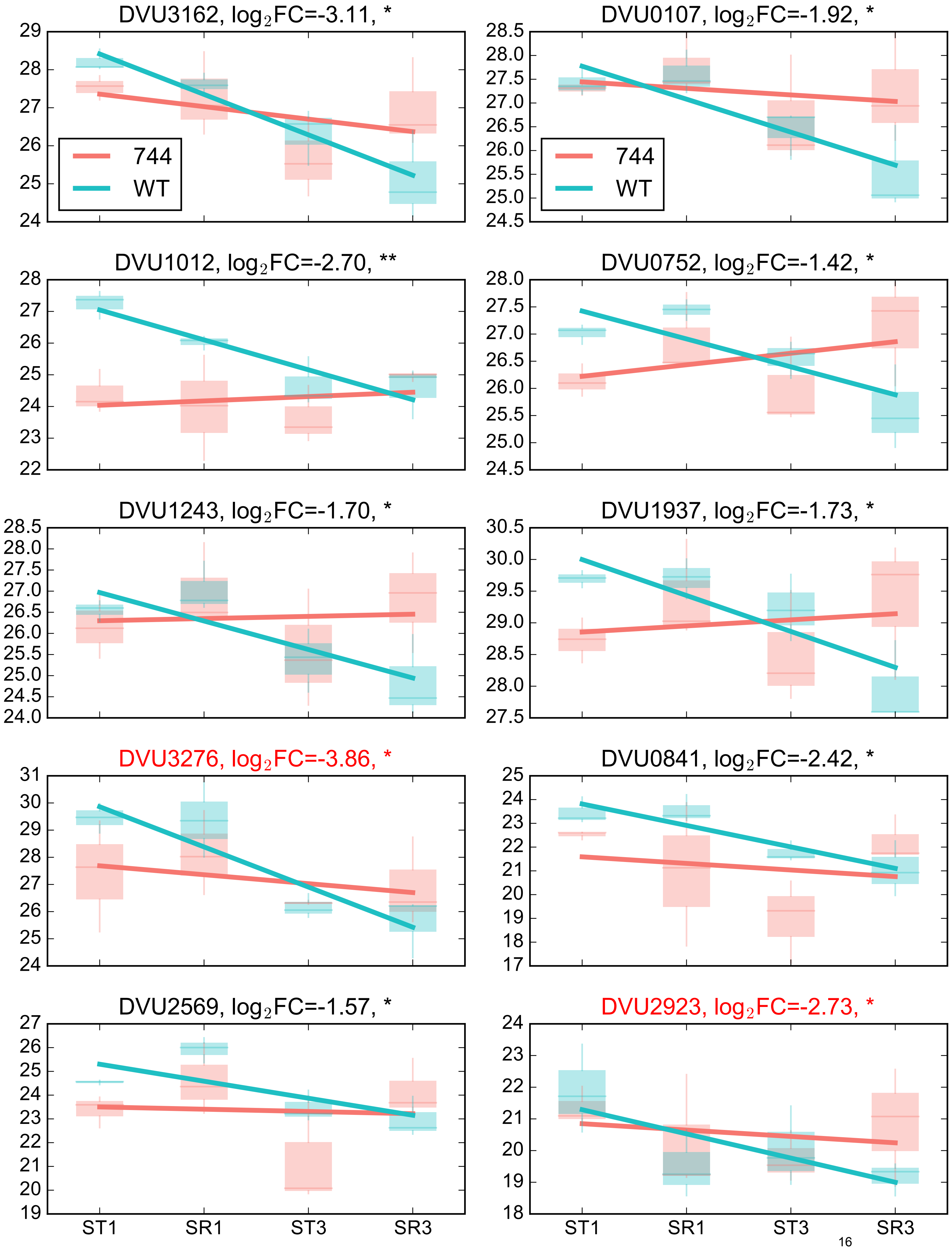
DVU3162, log2FC=-3.11, *
DVU0107, log2FC=-1.92, *
744
WT
744
WT
DVU1012, log2FC=-2.70, **
DVU0752, log2FC=-1.42, *
DVU1243, log2FC=-1.70, *
DVU1937, log2FC=-1.73, *
DVU3276, log2FC=-3.86, *
DVU0841, log2FC=-2.42, *
DVU2569, log2FC=-1.57, *
DVU2923, log2FC=-2.73, *
ST1
SR1
ST3
SR3
ST1
SR1
ST3
SR3

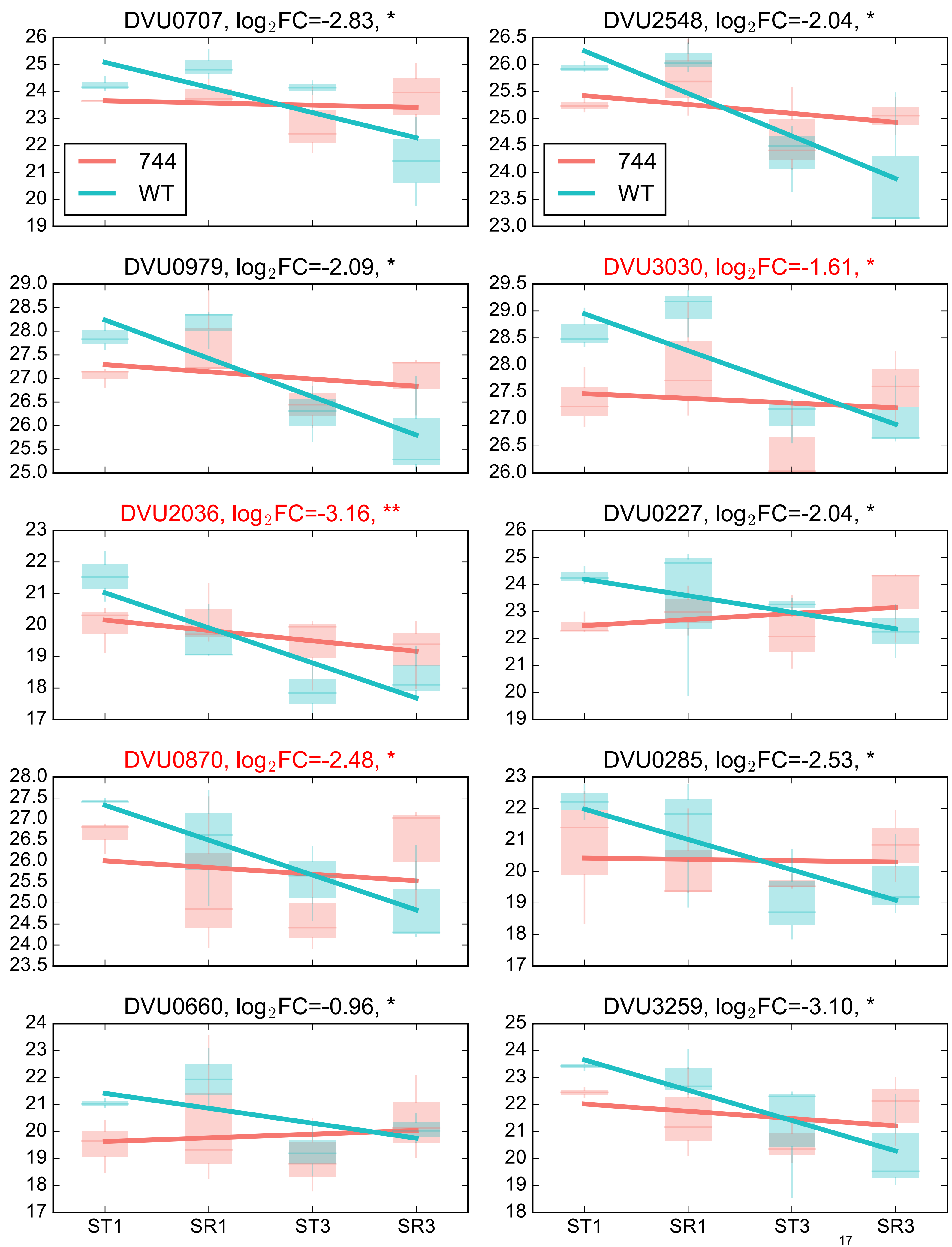
DVU0707, log2FC=-2.83, *
DVU2548, log2FC=-2.04, *
744
WT
DVU0979, log2FC=-2.09, *
DVU3030, log2FC=-1.61, *
DVU2036, log2FC=-3.16, **
DVU0227, log2FC=-2.04, *
DVU0870, log2FC=-2.48, *
DVU0285, log2FC=-2.53, *
DVU0660, log2FC=-0.96, *
DVU3259, log2FC=-3.10, *
ST1
SR1
ST3
SR3

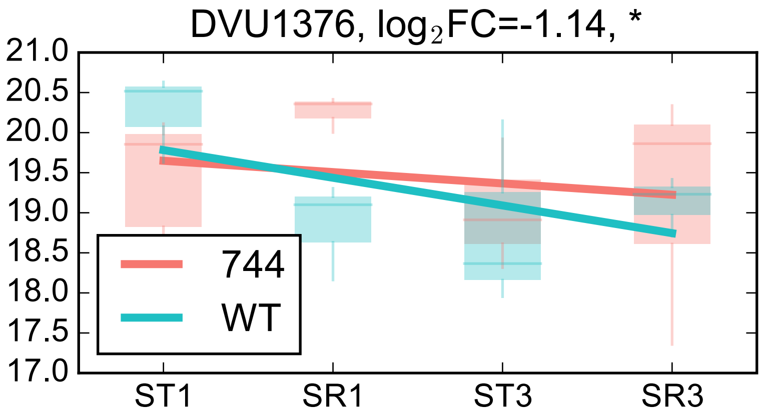
DVU1376, $\log_2$FC=-1.14, *
21.0
20.5
20.0
19.5
19.0
18.5
18.0
17.5
17.0
744
WT
ST1
SR1
ST3
SR3

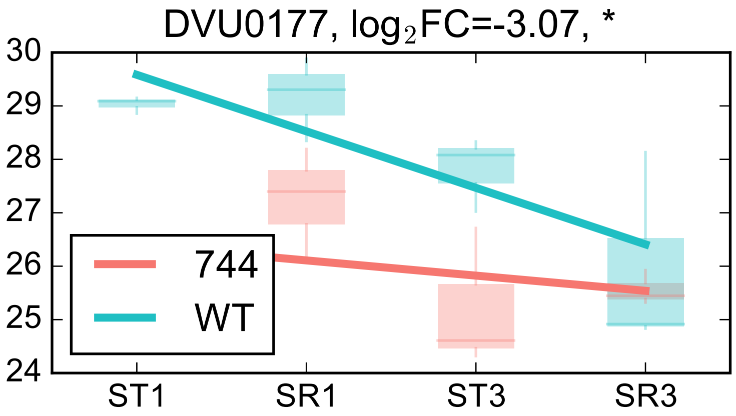
DVU0177, $\log_2$FC=-3.07, *
30
29
28
27
26
25
24
744
WT
ST1
SR1
ST3
SR3
