# Supplementary material for: Mechanism for microbial population collapse in a fluctuating resource environment
Source: Mol Syst Biol. 2017 Mar 20;13(3):919. doi: 10.15252/msb.20167058 (PMC5371734; doi:10.15252/msb.20167058)
Supplement: Supplementary file 2 — Expanded View Figures PDF [file MSB-13-919-s002.pdf]

Expanded View Figures

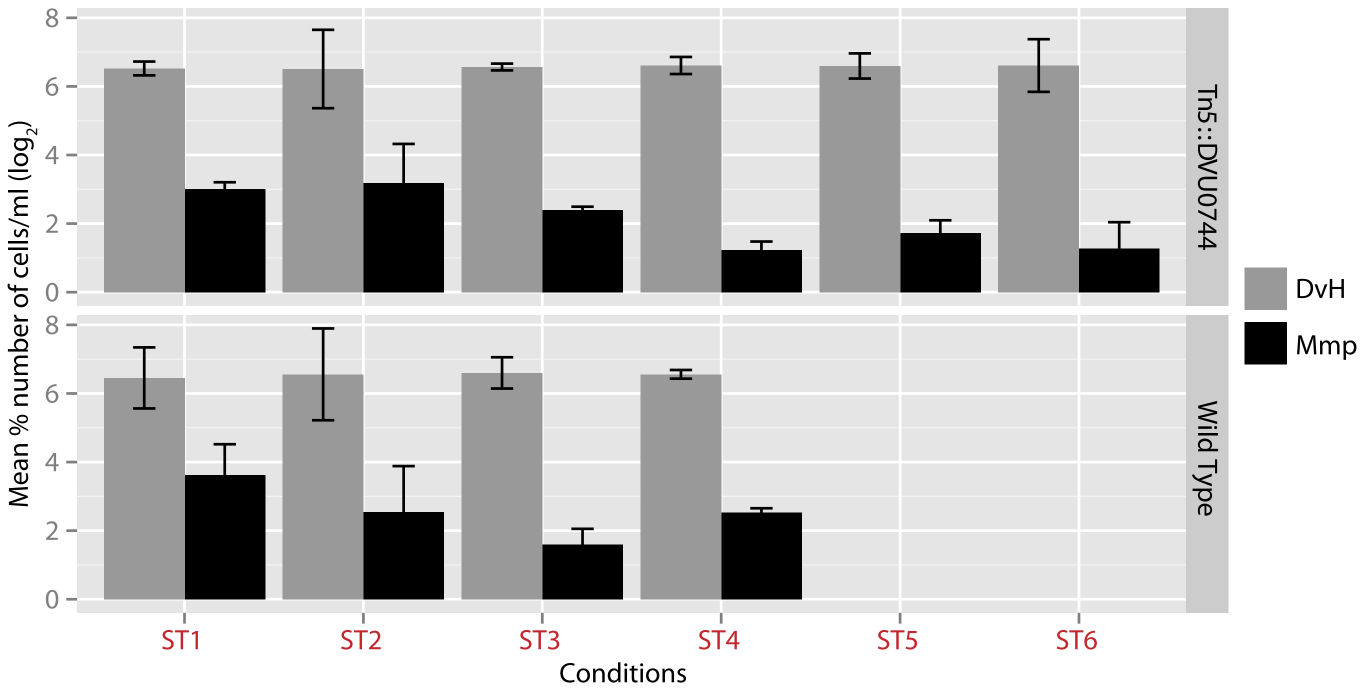

Figure EV1. Flow cytometry counts of Dv and Mm cells across all replicates and across multiple ST conditions.
Cell concentrations were calculated by dividing the number of events counted for each cell population by the volume sampled and reported as cell number per ml. Error bars correspond to standard deviation across 3 replicate measurements.

© 2017 The Authors

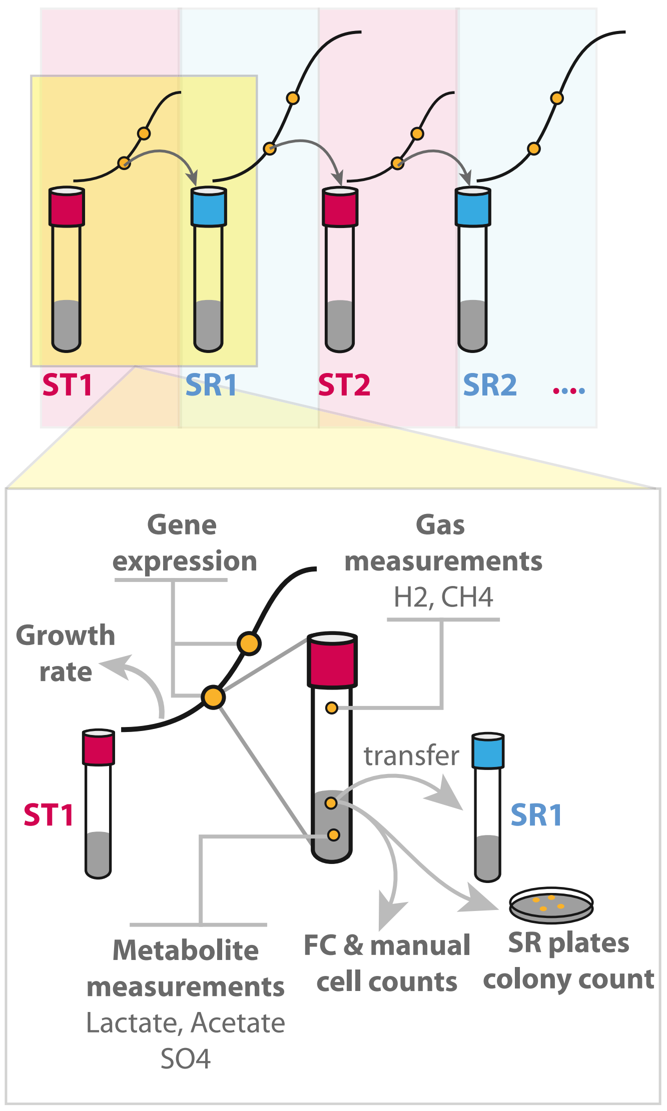

Figure EV2. Experimental workflow used in this study.

© 2017 The Authors

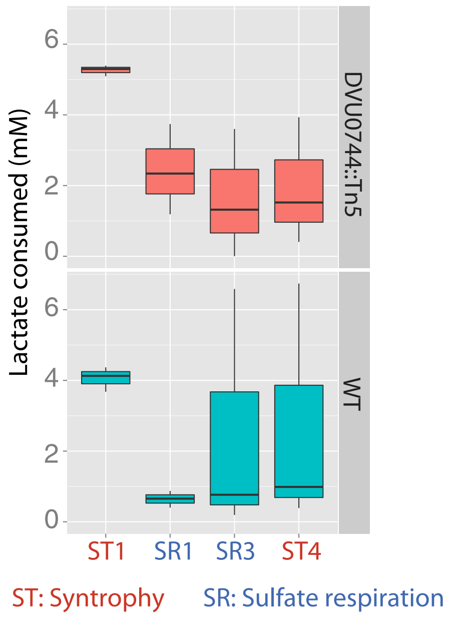

Figure EV3. Amount of lactate consumed by co-cultures during the transitions.
Boxplots for lactate consumption in wild-type (lower panel) and mutant (upper panel) co-cultures.

© 2017 The Authors

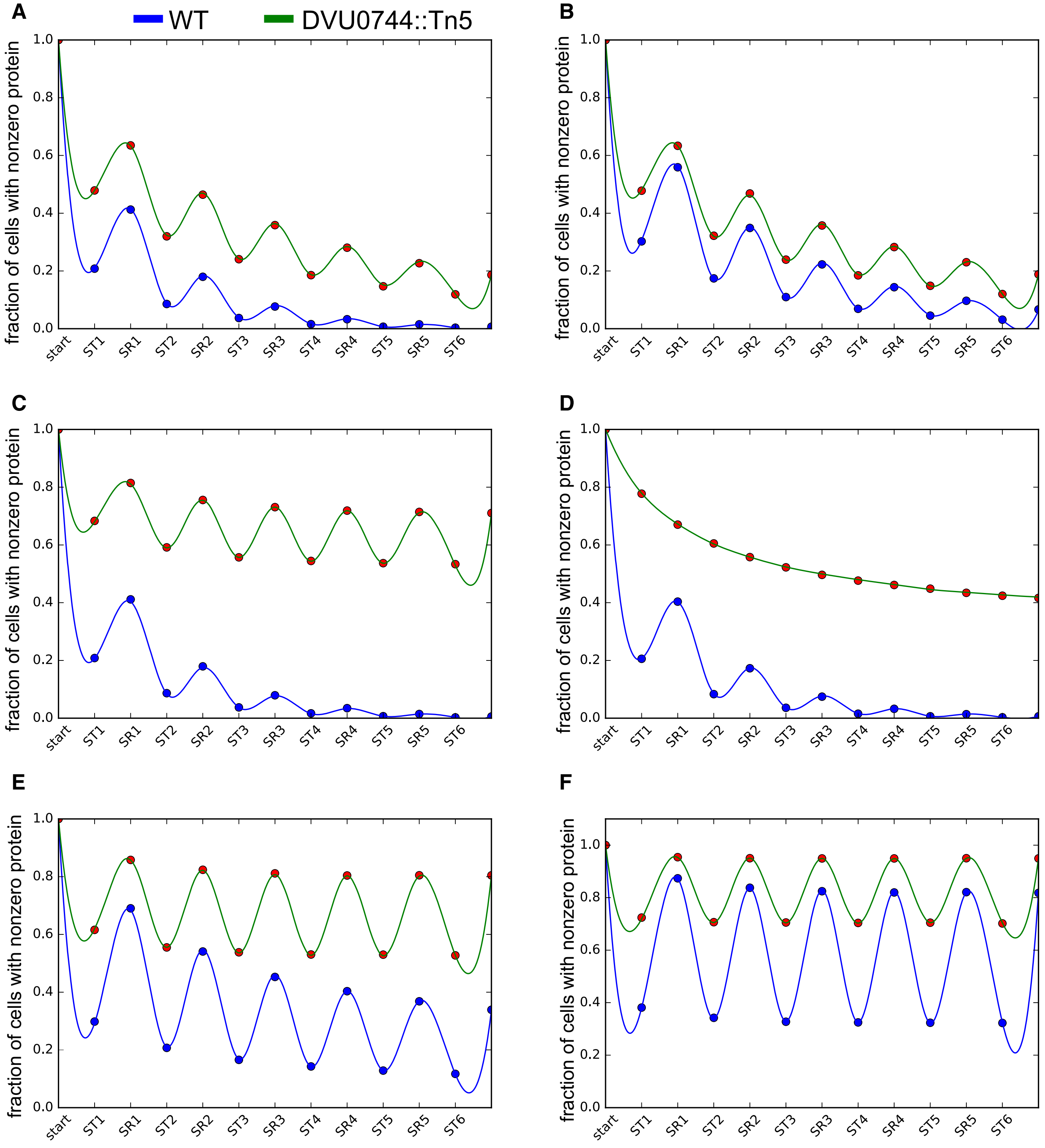

Figure EV4. The fraction of cells with nonzero copies of an essential protein for sulfate respiration is plotted over several transfers.

A–E Best estimates for starting points were obtained from the empirical fitting of Cai *et al* (2006), and our observed population-level transcript differences to produce the baseline figure in panel (A). Parameters were then modified as shown in Table EV13. In panel (B), we modified the degree of repression in the wild type to simulate a gene that was not as strongly repressed. In panels (C) and (D), we modified the degree of repression in the mutant to see the effect of increasing loss of regulation due to a regulatory mutation. Panel (C) shows that with twofold conditional repression, a protein will experience very little dilution across a cell population. In panel (D), there is no conditional repression at all, and the trajectory is a standard exponential decay. In panels (E) and (F), we explored the effect of translational efficiency by increasing the amount of proteins produced per burst. This simulation showed that very high translational efficiency can offset the effect of differential regulation, supporting the notion that conditionally essential low-abundance proteins are the most likely culprit in causing the extinction event.

 © 2017 The Authors
