# Supplementary material for: Mechanism for microbial population collapse in a fluctuating resource environment
Source: Mol Syst Biol. 2017 Mar 20;13(3):919. doi: 10.15252/msb.20167058 (PMC5371734; doi:10.15252/msb.20167058)
Supplement: Supplementary file 14 — Table EV12 [file MSB-13-919-s014.docx]

**Table EV12.** Microbial strains used in this study.

| Name | Strain name | Mutant Locus | Description | Organism |  | Reference |
| --- | --- | --- | --- | --- | --- | --- |
| Dv Wild type | Dv WT | - | ATCC 29579 | *Desulfovibrio vulgaris* Hildenborough |  | ATCC |
| Mm Wild type | Mm WT | - | - | *Methanococcus maripaludis* S2 |  | (Jones *et al*, 1983) |
| DVU0744::Tn5 | GZ12635 | DVU0744 | sigma-54 dependent transcriptional regulator / response regulator | *Desulfovibrio vulgaris* Hildenborough |  | (Zane & Wall, 2013) |
| DVU2802::Tn5 | GZ12895 | DVU2802 | transcriptional regulator, GntR family | *Desulfovibrio vulgaris* Hildenborough |  | (Zane & Wall, 2013) |
| DVU2275::Tn5 | GZ5190 | DVU2275 | sigma-54 dependent transcriptional regulator | *Desulfovibrio vulgaris* Hildenborough |  | (Zane & Wall, 2013) |

**References:**

Jones WJ, Whitman WB, Fields RD & Wolfe RS (1983) Growth and plating efficiency of methanococci on agar media. *Appl. Environ. Microbiol.* **46:** 220–226

Zane GM & Wall JD (2013) http://desulfovibriomaps.biochem.missouri.edu/mutants. Available at: http://desulfovibriomaps.biochem.missouri.edu/mutants
