# Supplementary material for: Mechanism for microbial population collapse in a fluctuating resource environment
Source: Mol Syst Biol. 2017 Mar 20;13(3):919. doi: 10.15252/msb.20167058 (PMC5371734; doi:10.15252/msb.20167058)
Supplement: Supplementary file 15 — Table EV13 [file MSB-13-919-s015.docx]

|  | a(SR, WT) | a(ST, WT) | a(SR, MUT) | a(ST, MUT) | b |
| --- | --- | --- | --- | --- | --- |
| A | 2.0 | 0.2 | 1.5 | 0.5 | 2 |
| B | 2.0 | 0.4 | 1.5 | 0.5 | 2 |
| C | 2.0 | 0.2 | 1.2 | 0.6 | 2 |
| D | 2.0 | 0.2 | 1.0 | 1.0 | 2 |
| E | 2.0 | 0.2 | 1.5 | 0.5 | 4 |
| F | 2.0 | 0.2 | 1.5 | 0.5 | 8 |

**Table EV13.** Model parameters modified for building Figure EV4.
